# An in-vivo study of photobiomodulation using 403 nm and 649 nm diode lasers for molar tooth extraction wound healing in wistar rats

**DOI:** 10.1007/s10266-021-00653-w

**Published:** 2021-09-07

**Authors:** Suryani Dyah Astuti, Age Sulistyo, Ernie Maduratna Setiawatie, Miratul Khasanah, Hery Purnobasuki, Deny Arifianto, Yunus Susilo, Kartika Anggraini Alamsyah, Suhariningsih, Ardiyansyah Syahrom

**Affiliations:** 1 Department of Physics, Faculty of Science and Technology, Universitas Airlangga, Surabaya, Indonesia 60115; 2 Biomedical Engineering Master Program, Faculty of Science and Technology, Universitas Airlangga, Surabaya, Indonesia 60115; 3 Biophysics and Medical Physics Research Group, Faculty of Sciences and Technology, Universitas Airlangga, Surabaya, 60115 Indonesia; 4 Department of Periodontology, Faculty of Dentistry, Universitas Airlangga, Surabaya, Indonesia 6011; 5 Department of Chemistry, Faculty of Science and Technology, Universitas Airlangga, Surabaya, Indonesia 60115; 6 Department of Biology, Faculty of Science and Technology, Universitas Airlangga, Surabaya, Indonesia 60115; 7 Faculty of Voccasional, Universitas Airlangga, Surabaya, Indonesia 60115; 8 Faculty of Engineering, Universitas Dr Soetomo, Surabaya, Indonesia 60118; 9 Department of Applied Mechanics and Design, Faculty of Mechanical Engineering, Universiti Teknologi Malaysia, 81310 Johor Bahru, Malaysia

**Keywords:** Tooth extraction, Health risk, Photobiomodulation, Diode laser, Wound healing

## Abstract

**Purpose:**

This study aims to examine the effects of red 649 nm 4 J/cm^2^ and blue 403 nm 8 J/cm^2^ diode laser treatment for post-extraction wounded healing in rats through histopathological and immunohistochemical analysis.

**Methods:**

Samples of 54 Wistar rats were divided into six groups: C- control group without treatment; C + wounded group without treatment; TB wound group with Povidone-iodine treatment; TD wounded group with doxycycline treatment; TLB wounded group with 403 nm diode laser treatment; and TLR wounded group with 649 nm diode laser treatment. Mandibular samples were observed for the number of lymphocytes and fibroblasts cells, new blood vessels formation, Interleukin 1β, and Collagen 1α expression level.

**Results:**

Based on the histopathological test results, red laser diode treatment significantly increased the number of lymphocyte, fibroblast cells and the formation of new blood vessels. Meanwhile, immunohistochemical tests showed an increase in the expression of the Colagen-1α protein which plays a role in the formation of collagen for new tissues formation after damage, as well as a decrease in Interleukin-1β expression level. Blue laser is also able to show a positive effect on wound healing even though its penetration level into the tissue is lower compared to red laser.

**Conclusion:**

The red diode laser 649 nm has been shown to accelerate the process of proliferation in wound healing post molar extraction based on histopathological and immunohistochemical test results.

## Introduction

Dental and oral diseases are major health problems in many countries affecting people their whole lives, causing increasing health risks, such as pain, discomfort, disability, and even in severe cases, leading to death [1]. One contributing factor of these diseases is tooth extraction process, a surgical procedure to remove a tooth from the dental alveolus (socket). This procedure leaves scar tissue in the tooth socket composed of cortical bone and causes severed periodontal ligaments wound [2]. Commonly, dental and oral diseases are not only caused by intentional extraction procedure but may also by unintended events such as trauma due to accidents or other cause. Based on statistics, increasing number of traffic accident rate may lead to higher cases of oral- and dental-related diseases. Many complications often occur in the wound healing process, such as alveolar osteitis (AO) after tooth extraction. AO or dry socket is a condition that causes pain for 1–3 days on the extraction site and around it, persists or increases after tooth extraction and does not dissipate after mild analgesics [3]. The pain is accompanied by partial or complete disintegration of the blood clot with or without halitosis.

Wounds can be classified into various degrees, i.e., acute wounds, chronic wounds, and complication wounds, whereas time is an important factor in the wound healing process [4, 5]. The process of wound healing in intentional or unintentional tooth extraction differs for each individual. Some factors considered in affecting the wound healing process, such as age, drug consumption, smoking habits, and bacterial infections, whose endotoxin results in a prolonged increase of pro-inflammatory cytokines, interleukin-1 (IL-1), and tumor necrosis factor alpha (TNF-α) production, thereby extend the inflammatory phase even (rarely) death [6].

Microorganisms that live in the oral cavity increase the risk of infection after tooth extraction. Post-tooth extraction infection is characterized by pain in the alveolus accompanied by suppuration, erythema, and edema with or without systemic fever [7]. Thus, prompt and appropriate treatment of wounds will greatly help reducing morbidity and mortality rates in patients with chronic wounds as well as reducing the financial burden and labor cost in prolonged time hospital management system [8]. The use of antibiotics is a common form of treatment to prevent infection in the wound healing process, including post-operative tooth extraction. Doxyxycline is a commonly used antibiotic against infections caused by Gram-negative and Gram-positive microorganisms [9]. Systemic treatment with antibiotics can eradicate infections causing bacteria as well as dental and oral diseases. The administration of antibiotics such as doxycycline is fairly effective in treating antimicrobial infections caused by Gram-negative and Gram-positive microorganisms, with great advantage as an anti-inflammatory agent by suppressing cytokines and chemokines production that cause inflammation [10] but has downside as it may lead to antibiotics resistance risk [11]. Therefore, alternative methods were used with antimicrobial photodynamic therapy (APDT) and photobiomodulation (PBM).

APDT utilizes a light source, photosensitizer (PS) and oxygen to produce Radical Oxygen Species (ROS). PS or PDT drug is a non-toxic chemical substance that will absorb light in its absorbance spectrum and through photooxidation [12]. The blue light spectrum of 405 nm shows conformity with the absorption spectrum of endogenous PS and exogenous PS, such as chlorophyll, curcumin and other types of PS [13, 14]. This condition will activate photophysical and photochemical reactions to produce ROS through energy transfer. ROS will inactivate pathogenic microorganisms, endo- and exotoxins [10].

PBM is a non-thermal method that utilizes light rays in the visible and infrared spectrum at low power. PBM in cells will stimulate cell growth, proliferation, and differentiation [15]. Based on observation effects in the wound healing process, there are several factors that contribute to the process, including fibroblast cells and keratinocyte cells activity [16]. Fibroblast cells will be stimulated in the proliferative phase. In this phase, fibroblast cells synthesize collagen as an extracellular matrix to form new tissue. Anthocyanins which are one of the flavonoid groups have the effect of increasing collagen synthesis by fibroblasts [17]. There is an increase in the number of fibroblast cells from day 3 to 14 post trauma, characterized by the replacement of the provisional matrix dominated by platelets and macrophages gradually replaced by fibroblast cell migration and deposition of extracellular matrix synthesis [4].

PBM and APDT are alternative therapies utilizing light sources such as laser which activates cell regeneration [18], light-emitting diode (LED) for antimicrobial [19], and broadband light in the visible and infrared spectrum with low power and energy density less than 50 J/cm^2^ [20]. This non-thermal therapy uses chromophore as a natural photosensitizing agent in photophysical and photochemical processes at various biological scales [21, 22]. Also, it is useful in reducing or relieving pain due to inflammation, regulating immunomodulation, as well as improving wound care and tissue regeneration [5, 23].

PBM therapy contributes to the wound healing process by stimulating cells to the regeneration stage, reducing pain, giving positive effects on inflammation, as well as accelerating proliferation and maturation phases accompanied by increasing tensile strength [14]. An injured body tissue generally experiences structural reduction and loss, both anatomically and functionally, so it is crucial for restoring function and structure as the effect of natural wound healing. Tissue repair involves several cells activity, such as epithelial cells, endothelial cells, as well as fibroblasts cells with their important role in the process [24]. Fibroblasts secrete multi-growth factors while the wound undergoes epithelialization. They are also actively involved in the forming of granular and synthetic tissue from a complex extracellular matrix after re-epithelialization. Hence, low-level laser light therapy is used to promote the biostimulation of fibroblasts and accelerate wound healing [25]. The use of a 635 nm laser with energy density 1 and 3 J/cm^2^ on Wistar rats results in cell proliferation within 24 h and demonstrates higher tensile strength in the wound compared to 809 nm laser application [26].

As the laser beam is exposed to the wound, the photon energy will increase Adenosine Tri-Phosphate (ATP) activity. Then, the excited ATP will trigger an immune reaction increasing pro-collagen and activate macrophages to produce fibroblast growth factor (FGF). The photon energy absorbed by the mitochondrial chromophore will double the activity of the mitochondrial respiratory chain which then increases the amount of ATP in the superficial tissue and brain. Furthermore, it will release nitric oxide (NO) and reactive oxygen species (ROS) as well as intracellular calcium and lead to wound healing and tissue necrosis prevention [27].

The results of PBM therapy with 632.8 nm diode laser at 3 mW/cm^2^, along with 5 or 16 J /cm^2^ on day 1 and day 4 after injury, caused a mitochondrial response for 1 up to 24 h. It was indicated by mitochondrial membrane potential (MMP) increase, as well as adenosine monophosphate (AMP), and ATP cycles, as well as intracellular calcium that produces homeostasis from injured cells and finally increases cell viability [17]. Moreover, PBM therapy stimulates wound healing with different processes, i.e., by increasing collagen production and deposition, stimulating proliferation of fibroblasts, and accelerating the formation of new blood vessels along with decreasing inflammation. Irradiation of 780 nm *InGaAsp* diode laser with energy density 0.5 and 1.5 J/cm^2^ on keratinocytes showed an increase in vascular endothelial growth factor (VEGF) and colagen-1α (Col-1) levels which indicates an increase in gene expression of keratin culture. While administration of photobiomodulation therapy with energy density 0.5 and 3 J/cm^2^ results in significant increase in cell metabolism compared to the absence of irradiation [17]. The results showed that 600–700 nm laser beam with energy density ranges from 0.5 to 4 J/cm^2^ resulting in the proliferation of cell cultures [28]. Another study stated that the use of a red laser with 4.2 J/cm^2^ energy density significantly improved wound healing in Wistar rats [29]. The use of a 637 nm laser with an energy density 4 J/cm^2^ and 50 mW intensity demonstrates a significant reduction in the swelling that occurred post molar tooth surgery [30].

Wound healing is a gradual process involving the activity of leukocytes and platelets [4]. Extraction and trauma to the tissue cause inflammation where in its early stages, the stimulus for injury or infection triggering the release of various inflammatory mediators, such as leukotrienes, prostaglandins, and histamine. Some cells will proliferate during tissue repair, including remnants of injured tissue (which attempt to perform remodeling into normal structures), vascular endosteal cells (forming new blood vessels and providing the nutrients needed during the repair process), and fibroblasts (the source of connective tissue which forms scar tissue to fill in the damage that regeneration process cannot repair) [4]. Such cell proliferation is motivated by proteins that act as growth factors, most of which are proteins that stimulate the survival and proliferation of certain cells which also lead to migration, differentiation, and other cellular responses [24].

Thus, the wound healing process is a challenging clinical problem required improvement, which means it is essential to have proper, effective, and efficient wound management [4, 24]. The post-extraction natural wound healing process is time-consuming and takes up to 16 weeks. This long duration is considerably due to the disturbances that may occur during the process, classified into two factors, namely local and systemic disturbance [7]. Local factors consist of infection, poor blood flow, foreign entities that can interfere with inflammatory mediator reactions, movement, type, size, and location. Systemic factors include age, nutrition, glucocorticoids, uncontrolled diabetes, and hematological abnormalities. Therefore, a mechanism is needed to accelerate the process of acute and chronic wound healing, one of which is using a *diode laser*.

In general, a diode laser is chosen for its ability to produce a monochromatic, coherent, and directional light beam. A diode laser is a photon source that activates the photosensitizer (PS) agent, causing molecular excitation (electronic transfer state) [31]. For the PS molecule to be excited, photon sources must emit a beam with a wavelength that is absorbable for it. The energy density of the diode laser exposure can be obtained by adjusting the time and power used [32].

This study analyzes the therapeutic effects of exposure on diode laser instrumentation in Wistar rats, with post-extraction wounds, through the immunohistochemical test for IL-1β, Col-1α levels, and the histopathological test on lymphocytes, fibroblasts, and the formation of new blood vessels. The analysis was carried out to find out whether red laser light exposure has a significant effect on wound healing, compared to the control positive, negative, antiseptic, antibiotic, and blue laser treatments. The data were analyzed by one-way ANOVA with hypothesis Ho: no difference between pre- and post-treatment, H1: there is difference between pre- and post-treatment. Decision: Ho rejected if: t calculation > t table, if *p* < α.

## Materials and methods

### Ethical approval

This study was approved by the Ethical Committee of the Faculty of Veterinary Universitas Airlangga with the reference number of 736-KE.

### Animals testing

The randomized in vivo treatment involved 54 male Wistar rats which had been acclimatized for 7 days and had met the following characteristics: (1) clinically healthy; (2) aged ± 12 weeks; and (3) ± 200 g weight. Ethical considerations used here with reference number: No. 736-KE, ethical clearance according to the treatment protocols in experimental animals. The stages done to experimental animals in this study include the acclimatization stage, the extraction stage, the treatment stage with the procedure of therapy administration, the euthanasia, and the preparations stage. The first molar tooth of the experimental animals was extracted, then treatments were given. This procedure was initiated by animal anesthesia with intramuscular injection on the femur. *Dexterous femur* from experimental animals was sprayed with alcohol and injected with ketamine hydrochloride dose of 40–100 mg/kg body weight (or equivalent with 0.2 ml) to get the anesthetic effect. After the rat was paralyzed (not responsive), the first molar was clamped with a miniclamp, then shaken to the right and left until the molar was extracted [33].

### Light source

The blue diode laser (Sony) emitted 403 nm wavelength with output power 27.65 ± 0.01 mW and 0.152 ± 0.009 cm^2^ focus spot area, 44 s irradiation time and 8 J/cm^2^ energy density [34]. The red diode laser (Sony) emitted 649 nm wavelength with output power of 15.42 ± 0.08 mW and 0.164 ± 0.009 cm^2^ focus spot area, 42 s irradiation time and 4 J/cm^2^ energy density [17]. The diode laser characterization was done by Jasco CT-10 monochromator. Temperature measurement during irradiation process showed temperature stability in 32 °C ± 0.20 °C.

### Treatment procedures

Wistar rats were divided into six groups: (1) C- (K-) Group as the healthy control group without treatment; (2) C + (K +) Group as the wounded group without treatment; (3) TB Group as the wounded group with Povidone-iodine (Brand Betadine, PT. Mahakam Beta Farma, Indonesia) treatment, (4) TD Group as the wounded group with doxycycline treatment, (5) TLB Group as the wounded group with 403 nm diode laser treatment; and (6) TLR Group as the wounded group with 649 nm diode laser treatment. Two different laser irradiations were used, i.e., (i) 403-nm diode laser with 44 s irradiation time and energy density of 8 J/cm^2^ and (ii) 649 nm diode laser with 42 s irradiation time 4 J/cm^2^ energy density. These therapies were conducted by irradiation procedure perpendicular to the wound area [34]. Antibiotic treatment using 100 mg doxycycline was diluted with distilled water to obtain a concentration of 0.1%. Next, a micro-brush was immersed in the doxycycline solution, then applied to the wound area. In the TB Group therapy, a micro-brush was immersed in the Povidone-iodine, then applied to the wound area. Samples extraction and euthanasia process were taken 24 h after treatment. The mandibular tissue was extracted and incised with a scalpel, then put into 10% neutral formalin-buffered solution in urine pot and finally stored until 3 days. Furthermore, histopathological and immunohistochemical observations were carried out.

### Histopathological observation

Immunohistochemistry sample preparations include decalcification, embedding, de-paraffination, and immunostaining process. Mandibular slices were fixed using 10% buffer neutral formalin (BNF) solution, then trimmed and inserted into an embedding cassette made of plastic. Next, the alcohol dehydration process was carried out using 70%, 80%, 90% alcohol, absolute alcohol I, and absolute alcohol II, which then were purified using xylol I and xylol II. Then, the molding or *paraffinization* process was carried out using paraffin I and paraffin II. The slides were inserted into a molding device filled with half-volume paraffin and placed vertically or horizontally so that the slices transversely positioned and attached to the paraffin base. After the slides started to freeze, the paraffin was added again until the molding device was full and allowed them to harden. After that, they were cut into 5 μm thickness using a microtome. The ribbon shape of the (tissue) pieces was stretched using warm water at 45 °C. The slices were placed on top of object glass, then dried overnight in an incubator at 60 °C. Lastly, they were stained with Hematoxylin–Eosin (HE) and Masson Trichrome staining to observe the fibroblast tissue [34].

Hispatological observations on tissue samples were conducted on day 1, day 2, and day 3. The parameters of this histopathological examination include lymphocyte cells that represent the level of inflammation, the number of fibroblasts cells, and the formation of new blood vessels which expresses the inflammatory phase and the proliferative phase in wound healing, respectively [35]. Observations of the above parameters were done by microscope, with 400 × magnification and 8 × field of view.

### Immunohistochemical preparations

Immunohistochemical observation used in this study was indirect immunohistochemical method to examine specifically on Interleukin 1β (IL-1β) protein and Collagen 1α (Col-1α) expression [36]. Immunohistochemical sample slides were prepared by *de-paraffination* using xylol as cleaning fluid, applied three times with 5 min duration in each sample, then they were rehydrated using 100%, 96%, 80%, and 70% alcohol for 4 min using running water. Next, endogenous blocking was carried out using 0.3% peroxide for 10 min, then cleaned again under running water. After that, the antigen retrieval de-cloaking chamber was used, the slides were cooled and cleaned using phosphate-buffered saline (PBS) (pH 7.4) for 5 min and blocked with an excel block for five minutes. After administrating the primary antibody, the samples were left for about 60 min then cleaned with PBS (pH 7.4) for 5 min again, followed by excel blocking, and then let them stand for 20 min [37].

Next, Chromogen 3,3′-Diaminobenzidine (DAB) and the addition of buffer substrate were performed, in this step, 1 drop of DAB added to 1 ml of the substrate, then samples slides were cleaned again under running water. Lastly, counterstain with Hematoxylin was carried out for 1 min, followed by dehydration with alcohol and cleansing of xylol. The immunohistochemical test conducted in this study aims to determine the expression level of inflammatory mediators that stimulate periodontal tissue regeneration in wound healing, including IL-1β and COL-1α expression. Observations on immunohistochemical sample slides were carried out using light microscope with 4 × and 10 × magnification. The expression level of each inflammatory mediator was obtained by manually recording its expression number.

### Data analysis

The observation data results of lymphocytes cells, fibroblast cells, blood vessels, Interleukin-1β, and Collagen-1α measurement were performed statistically to show the significant difference among treatments, taking in advance the cut-off level for statistical significance at α = 0.05.

## Results

### Histopathological observations results

**Fig. 1:**
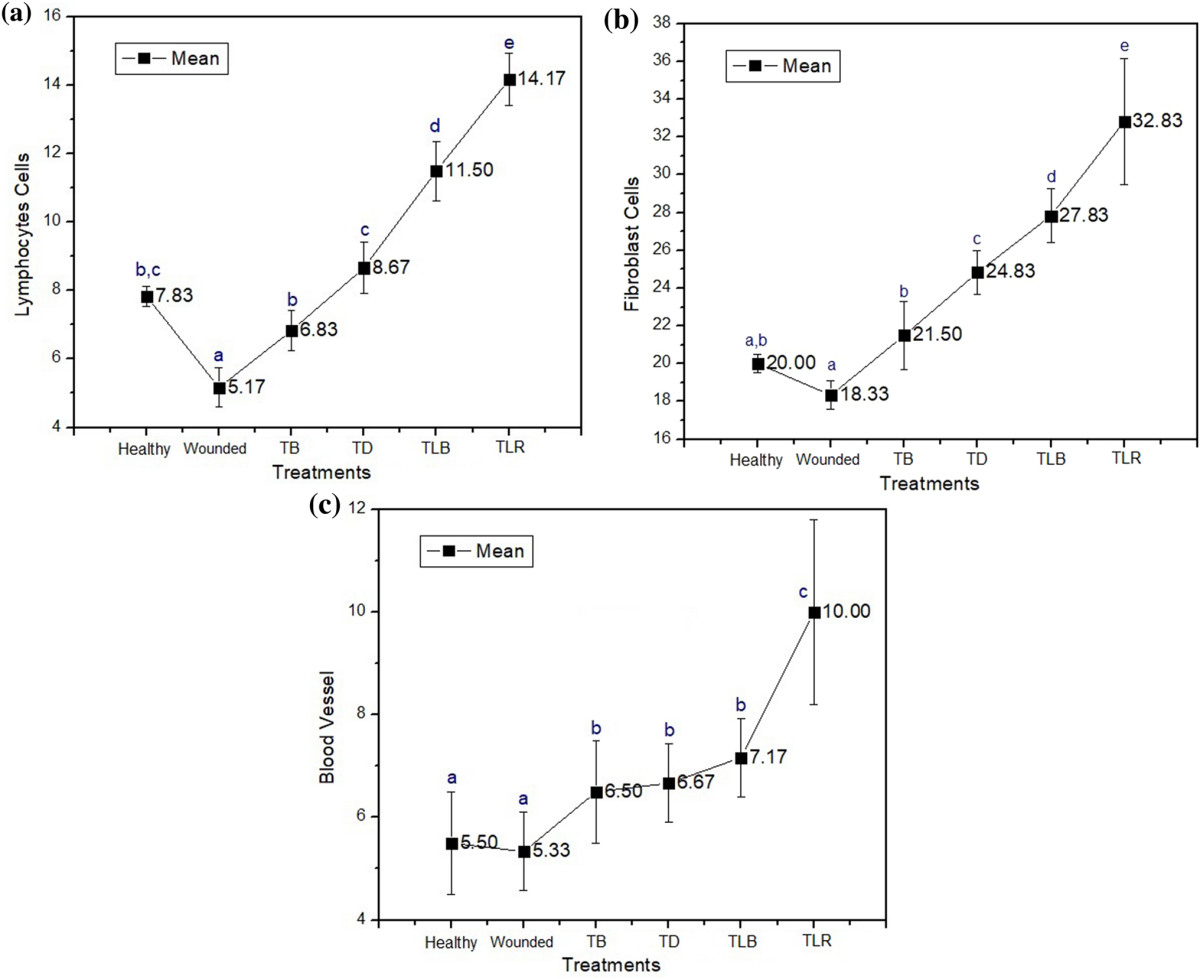
Comparison graphs of the mean and significant differences of each treatment for all sample groups including the number of lymphocytes (**a**), fibroblasts (**b**), and blood vessels (**c**). *Same letter in group (**a, b, c, d, and e**) defines non-significant results

The results of histopathological observations carried out on day 1, day 2, and day 3 are shown in Fig. 1. In the wound healing process, the inflammatory phase usually takes place on the first day of injury until approximately the fourth day. Fibroblasts which produce a new extracellular matrix are more active in synthesizing matrix components in response to injury. Migration and proliferation of fibroblasts in the wound area will increase collagen synthesis. Collagen will provide strength and integrity for the wound tissue to heal properly [38].

The observation results showed that the number of lymphocyte cells in all treatment group significantly different (*p* = 0.00) compared by red laser treatment. The lymphocyte cells in wounded Wistar rat during inflammation were lower than the healthy rat on day 1 to day 3. A significant difference occurred on day 3 when the lymphocyte cells of the wounded rat were 4.5 ± 0.5 cells, which are less than the healthy one (8 ± 1 cells). In all treatment groups, it was found that the number of lymphocyte cells in the wounded rat was less than the Povidone-iodine treatment group, the doxycycline treatment group, the blue laser group, and the red laser treatment group. In the red laser treatment group, the highest number of lymphocyte cells was significantly observed (*p* = 0.00) compared to other groups, with a range of 10.5 ± 0.5 to 13.5 ± 0.5 cells, which showed significant differences (*p* = 0.00) occurring on day 1 of 15 ± 1 cells and a mean of 14.17 ± 1.47 cells.

**Fig. 2:**
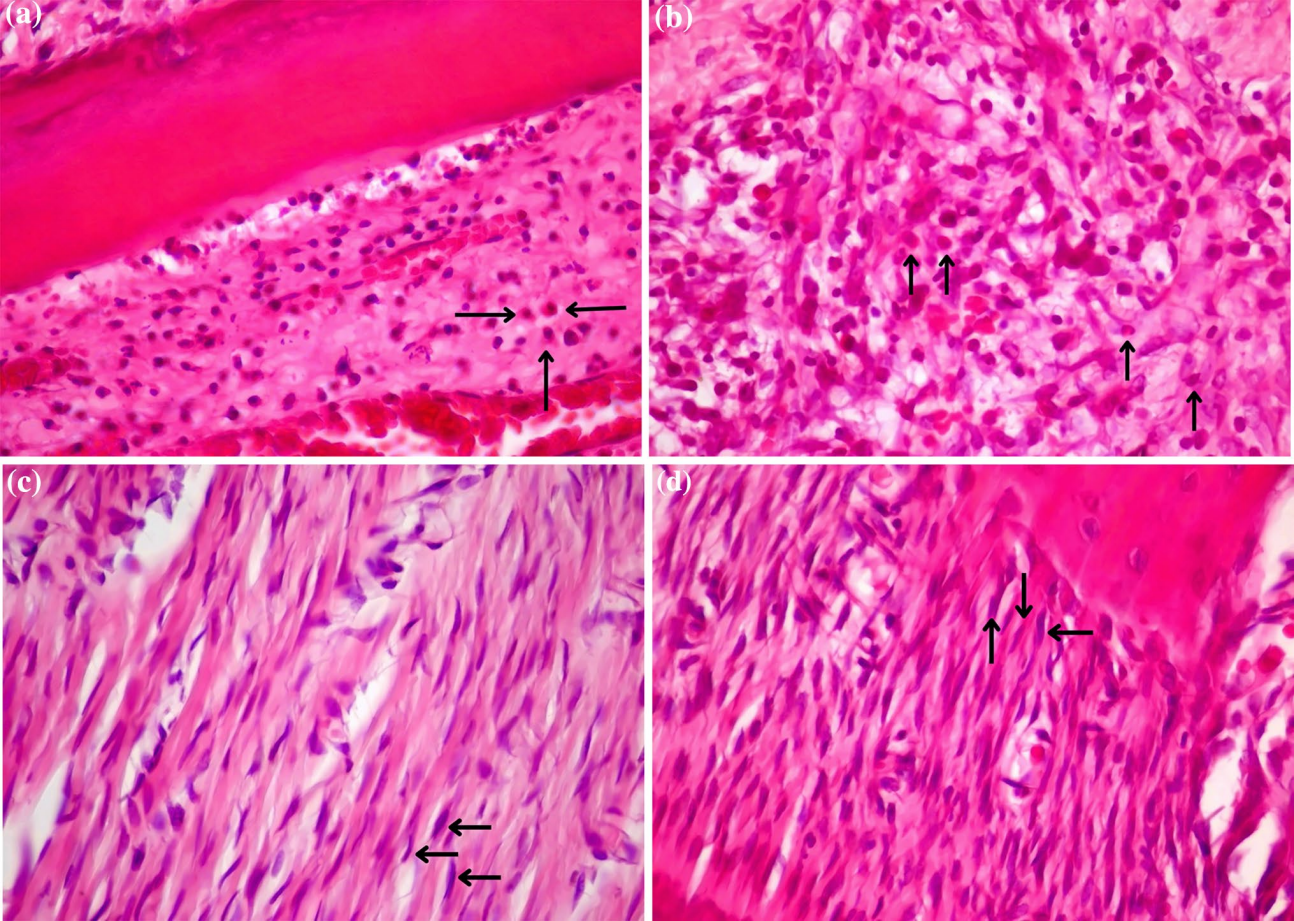
Histological slides **a** number of lymphocytes in the wounded Group and **b** number of lymphocytes in the Red Laser Treatment Group and **c** the number of fibroblasts in the wounded Group and **d** the number of fibroblasts in the Red Laser Treatment Group (observation with 400 × magnification and 8 × field of view)

Observation of the cell proliferation phase was carried out by calculating the number of types of cells that support the wound healing process, i.e., fibroblasts on day 1, day 2, and day 3 after the injury. Figure 2 shows the histological sample slides for (a) the number of lymphocytes in the wounded Group and (b) the number of lymphocytes in the Red Laser Treatment Group and (c) the number of fibroblasts in the wounded Group and (d) the number of fibroblasts in the Red Laser Treatment Group, with 400 × of magnification and 8 × of a field of view.

The observation results showed that the number of fibroblast cells in control and all treatment group was significantly different (*p* = 0.00) compared to red laser treatment. The wound healing process of the group was lower than that of the healthy group on day 1 until day 3. A significant difference (*p* = 0.00) occurred on day 2 when the number of fibroblasts in the wounded rat was 17.5 ± 0.5 cells, which are significantly less (*p* = 0.00) than the healthy rat (20 ± 1 cells). In all treatment groups, it was found that the number of fibroblast cells in the wounded rats was less than fibroblast cells found in the Povidone-iodine treatment group, the doxycycline treatment group, the blue laser group, and the red laser group. In the red laser treatment group, the number of fibroblast cells was higher compared to all treatment groups which showed a range of 29.5 ± 1 to 35 ± 1 cells, with a significant difference (*p* = 0.00) occurring on day 3 of 35 ± 1 cells and a mean of 32.83 ± 3.31 cells.

**Fig. 3:**
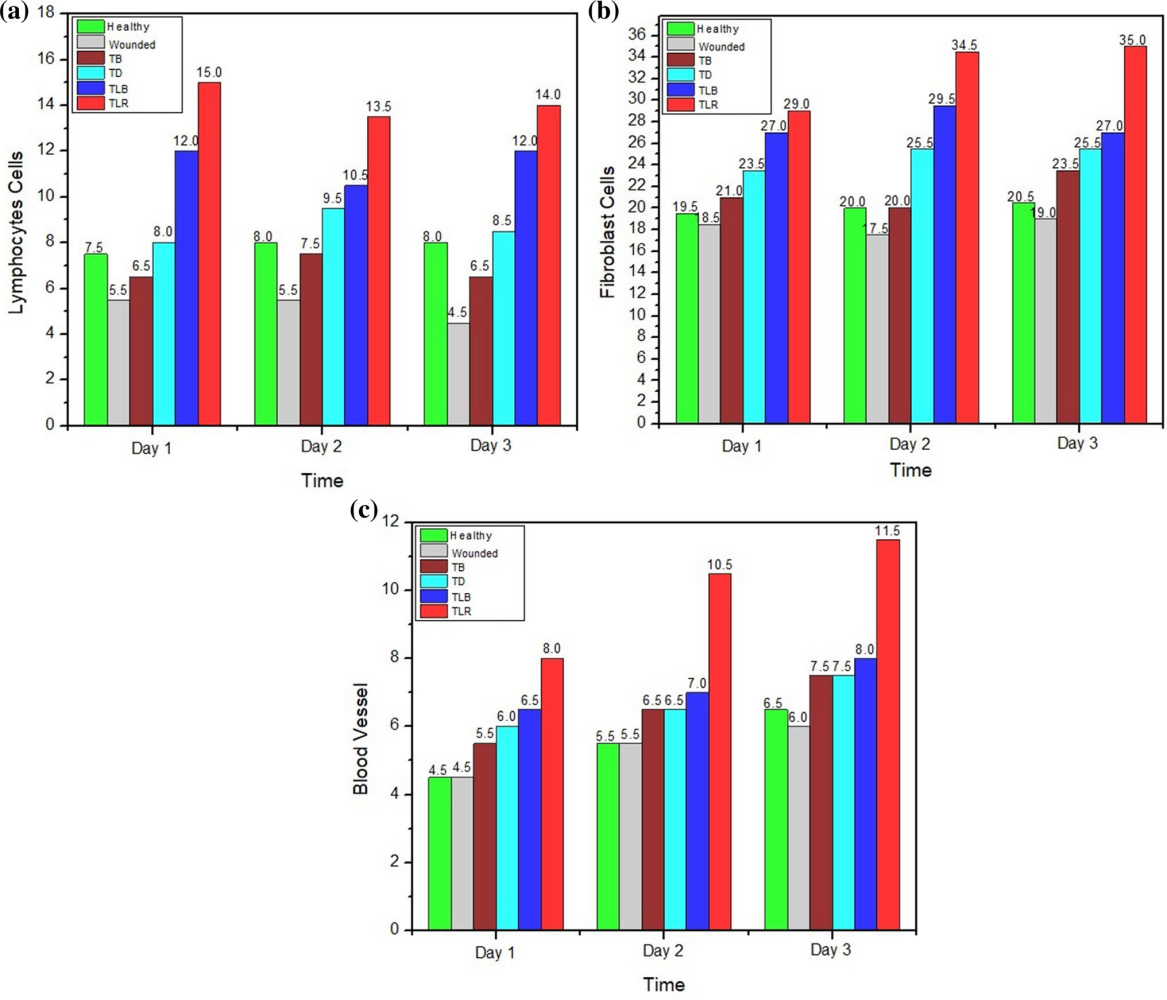
Comparison in the numbers of **a** lymphocytes, **b** fibroblasts, and **c** formation of blood vessels between treatment groups

The observation results for the number of blood vessels as the indicator of the wound healing process were carried out on day 1, day 2, and day 3 after injury. Overall, the results indicated a significant difference (*p* = 0.00) in the number of blood vessels between the treatment groups and the control groups compared to red laser treatment group. In all treatment groups, it was found that the number of blood vessels in wounded and healthy rats was less than the Povidone-iodine treatment group, the doxycycline treatment group, the blue laser treatment group, and the red laser treatment group. In the red laser treatment group, the number of blood vessels was the highest compared to all other groups with a range of 8 ± 1 to 11.5 ± 0.5 cells and a significant difference (*p* = 0.00) occurred on day 3 which showed 11.5 ± 0.5 cells and a mean of 10.00 ± 1.67 cells. Figure. 3 shows diagrams of the number of lymphocytes, fibroblasts, and blood vessels for each treatment group with various time ranges.

### Immunohistochemical test results

**Fig. 4:**
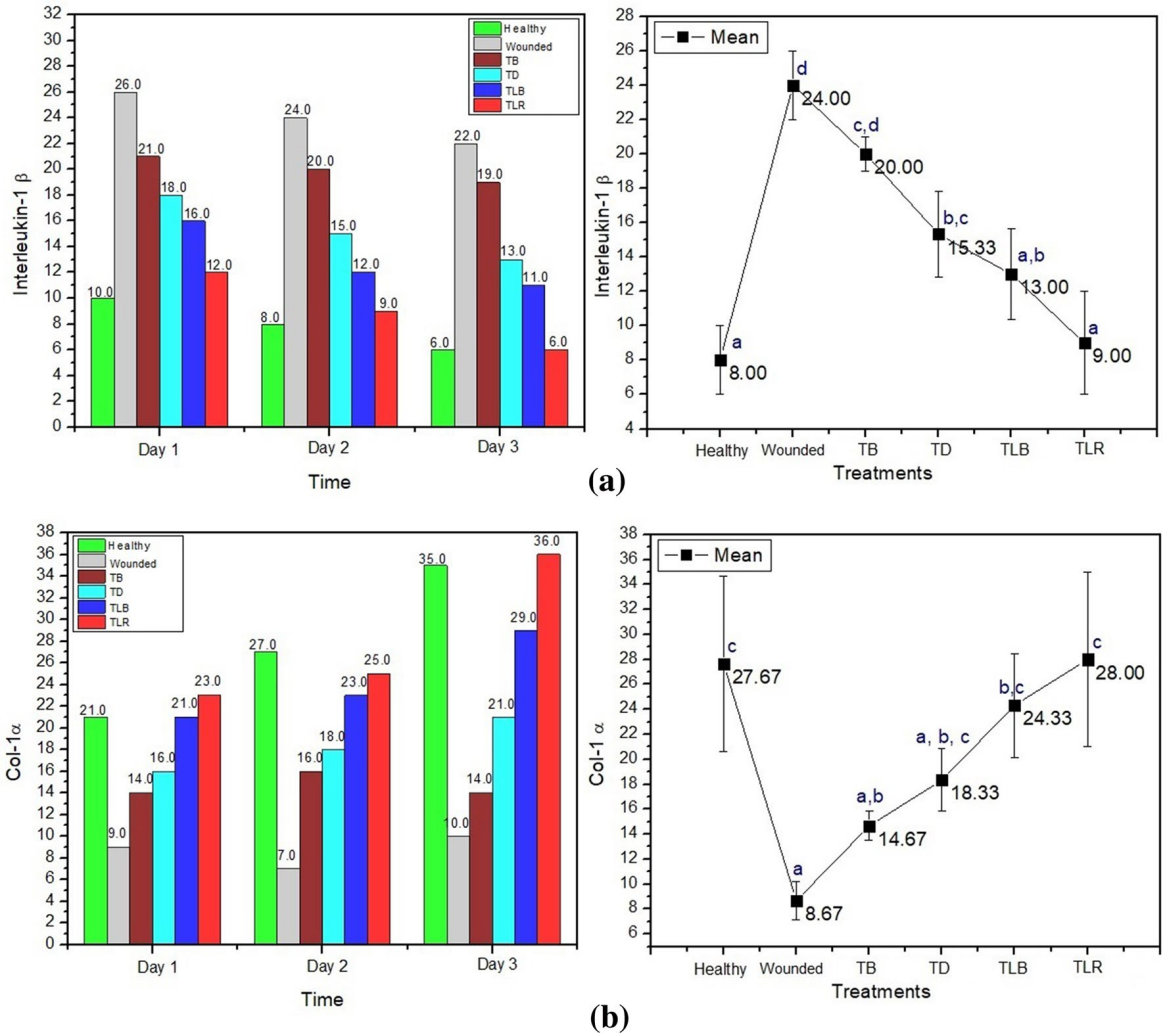
Mean graphs of **a** IL-1β with its comparison numbers in each treatment group and **b** Collagen-1α between treatment groups with its comparison numbers in each treatment group. *Same letter in group (**a, b, c, d, and e**) defines non-significant results

The test results showed that the IL-1β protein expression of the wounded rat group was higher than the healthy group from day 1 to day 3. Meanwhile, the expression of IL-1 β in wounded rats was higher than the other treatment groups with α = 0.05 significance level. In all treatment groups, the amount of IL-1β on day 1 to day 3 was below the range for the wounded groups. In the Povidone-iodine treatment group, the highest expression recorded is 20 ± 1 cells compared to other treatment groups: 15.33 ± 2.52 cells in the doxycycline group; 13 ± 2.65 cells in the blue laser group; and 9 ± 3 cells in the red laser group. The graphs of mean and significant differences in each treatment group are captured in Fig. 4**.**

**Fig. 5:**
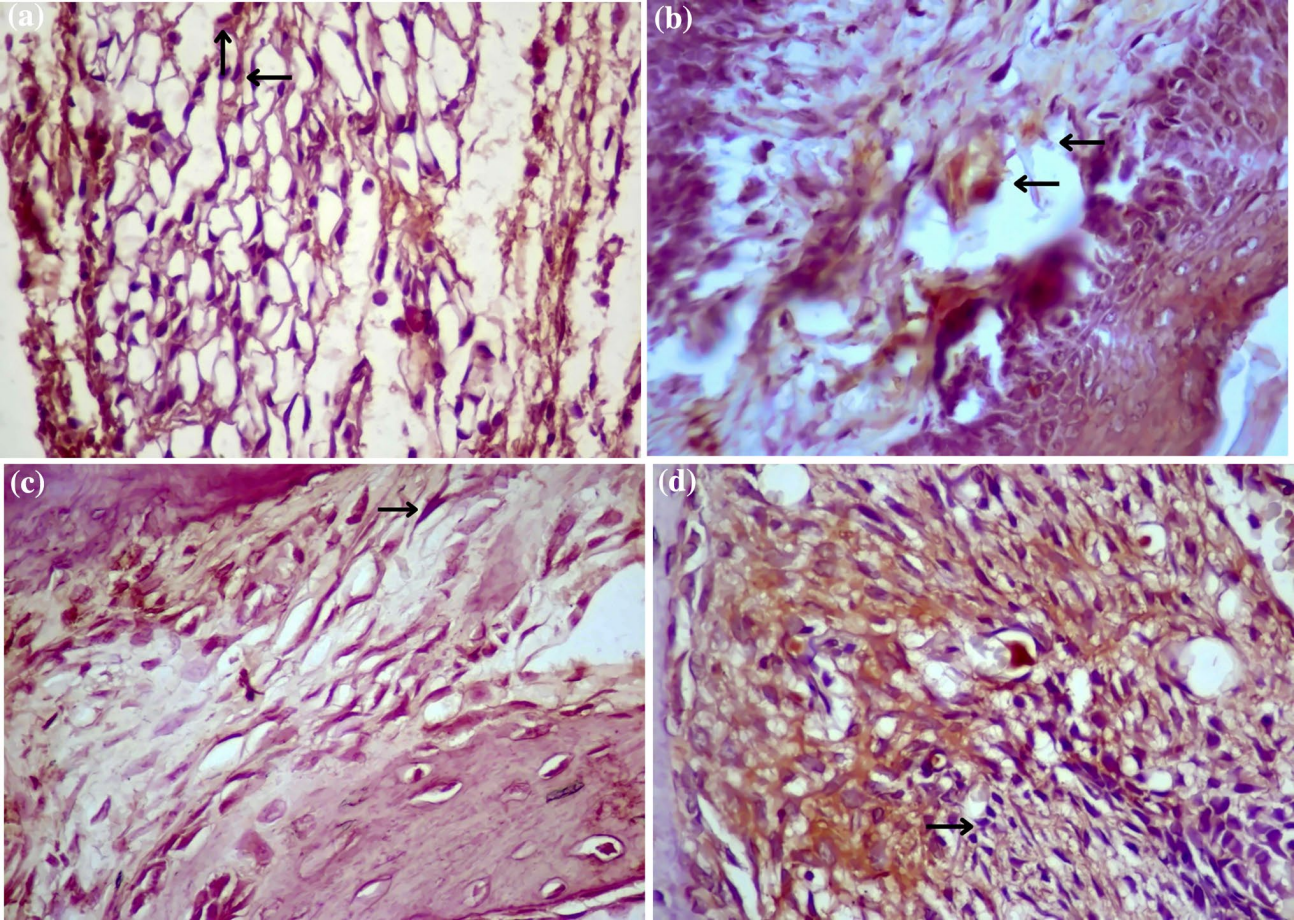
Comparison of Interleukin-1β in the wounded group (**a**), Red Laser (**b**) as well as comparison of Collagen-1α in the wounded Group (**c**), and Red Laser (**d**), with Immunohistochemical test and 400 × magnification with 8 × field of view

The red laser treatment group had expression level of IL-1β which is not significantly different (*p* = 0.99) when compared to the healthy group and the doxycycline treatment group (*p* = 0.05), and significantly different (*p* = 0.00) when compared to the wounded group, the Povidone-iodine treatment group (*p* = 0.00), and the blue laser treatment group (*p* = 0.03), occurred on day 2 and day 3. The expression level of IL-1β in photobiomodulation therapy using red laser showed a significant difference (*p* = 0.00 < α = 0.05) with decline starting from day 1 compared to other therapy groups. On day 3, the wounded group and the red laser photobiomodulation group showed different values (*p* = 0.05), but not significantly different. Figure. 5 exhibits the expression levels of IL-1β belonged to the wounded Wistar rat group and the red laser photobiomodulation group.

The results showed that the red laser treatment group had expression Collagen-1α which is not significantly different (*p* = 0.99) when compared to the healthy group and the doxycycline treatment group (*p* = 0.07), and significantly different when compared to the wounded group (*p* = 0.00), the Povidone-iodine treatment group (*p* = 0.02), and the blue laser treatment group (*p* = 0.00), on day 1 until day 3. The number of Collagen-1α in the red laser treatment group was 28.00 ± 7.00, higher than the wounded Wistar rat group, the Povidone-iodine treatment group, and the doxycycline treatment group, occurred on day 2 and day 3. Figure. 4a, b depicts the comparison number of Interleukin-1 β and collagen for each treatment groups over 3 days period.

The results showed an increase in Collagen-1α which indicated wound repair process occurred in both red laser treatment and other treatment groups. Based on the histopathological and immunohistochemical observation results, there was a significant difference between the red laser treatment and other treatments, except for Interleukin-1β in the healthy group (*p* = 0.99), in the doxycycline group (*p* = 0.05), as well as level of Collagen-1α (*p* = 0.99) in the healthy group and the doxycycline group (*p* = 0.07). Table 1 shows the difference in the effect of the 649 nm red laser treatment compared to other treatments based on the results of histopathological and immunohistochemical tests.

**Table. 1:** One-way ANOVA statistical analysis of red laser treatment compared with other treatments based on the results of histopathological and immunohistochemical tests

Groups		Histopathology						Immunohistochemistry			
		Lymphocytes Cells		Fibroblast Cells		Blood Vessels		Interleukin-1β		Collagen-1α	
		Mean	*p*	Mean	*p*	Mean	*p*	Mean	*p*	Mean	*p*
Red Laser	Healthy	8.00 ± 0.89	0.00	20.00 ± 1.26	0.00	5.33 ± 1.03	0.00	8.00 ± 2.00	0.99	27.66 ± 7.02	0.99
	Wounded	5.16 ± 0.75	0.00	18.33 ± 1.03	0.00	5.16 ± 0.75	0.00	24.00 ± 2.00	0.00	9.66 ± 0.58	0.00
	Povidone-iodine	6.83 ± 0.75	0.00	21.5 ± 1.87	0.00	6.33 ± 0.81	0.00	20.00 ± 1.00	0.00	15.33 ± 1.15	0.02
	Doxycycline	8.66 ± 0.81	0.00	24.33 ± 1.47	0.00	6.67 ± 0.81	0.00	15.33 ± 9.08	0.05	18.33 ± 2.52	0.07
	Blue Laser	11.16 ± 0.75	0.00	27.83 ± 2.40	0.00	7.16 ± 0.75	0.00	13.00 ± 2.65	0.03	24.33 ± 4.16	0.01

## Discussion

PBM is an alternative therapy that utilizes light sources, such as laser, LED, and broadband light in the visible and infrared spectrum, with low power and low energy density [34]. Lymphocytes cells that play role in the immune system will increase in the inflammatory phase as they migrate to the wound area on the first day, then the number will peak on the third to sixth days, finally on the seventh day their level drop [35]. During inflammation, this cell has functions as the humoral and cellular response, becoming one of the first cells to reach the wound area. Its main function is removing infection and cleaning the cellular matrix debris from foreign substances binding antigens, then it will be activated to secrete lymphokines, one of which is interferon gamma (IFN-γ) [36]. Lymphokines help the stimulation and activation of macrophages in the phagocytosis. Macrophages are responsible for phagocytic injury and polymorphonuclear (PMN) cells that have undergone apoptosis. The activated macrophages will release cytokines, namely IL-1 and TNF, which then activate lymphocytes [37]. Lymphocytes and macrophages stimulate each other persistently to eliminate the antigens so that the fibroblasts can form a strong tissue [4, 36].

The observation results (see Table 1) resulted in *p* = 0.00 which indicates the hypothesis H_o_ is rejected and H_1_ is accepted, which means that there is a significant difference between the red laser treatment and the other treatment groups. A significant increase (*p* = 0.00) of lymphocytes in the red laser treatment group is shown on day 3 after the injury compared to the number of lymphocytes observed from the other treatment groups. In most cases, antibiotics drops (doxycycline) and antiseptic (Povidone-iodine) are used by the public to heal wounds. The study in lymphocyte cells and fibroblast cells showed that the red laser treatment had the highest mean value which was significantly different from other treatments. The number of lymphocytes increases since day 1 after laser beam exposure [36].

In the wound healing process, there are several contributing factors, i.e., fibroblast cells, and keratinocyte cells. Fibroblasts will be stimulated in the proliferation phase to synthesize collagen as an extracellular matrix to form new tissue [28]. A significant increase (*p* = 0.00) in the number of fibroblasts from day 3 to 14 after trauma is indicated by the replacement of the provisional matrix dominated by platelets and macrophages gradually being replaced by fibroblast cell migration and deposition of extracellular matrix synthesis [38]. According to fibroblast cells observation results, there was a significant increase (*p* = 0.00) in the red laser treatment group on day 3 after the injury compared to the number of fibroblasts from the other treatment groups. This signifies (*p* = 0.00) that the red laser treatment accelerates wound healing process better than antibacterial and antibiotics treatment. Also, the Tukey post hoc test showed that the highest number of fibroblasts (32.83 ± 3.31) is shown in the red laser treatment group, different significantly (*p* = 0.00) from the other groups. This result supports the previous finding that laser exposure to the wound tissue increases the number of fibroblasts starting on the second day [36], with the fibroblasts increase caused higher tensile strength of the tissue [23].

Wound defined as a condition when biological tissue loses its normal function both in anatomy and function. Naturally after wound occurred, healing process will follow. The healing process includes homeostatic, inflammation, proliferation, and remodeling, which will immediately occur after the injury happened to biological tissue [39].

In the wound-healing phase, the formation of blood vessels is important for the re-epithelialization process. Angiogenesis occurs in response to angiogenic factors that stimulate new capillaries as the result of the venules’ growth. Endothelial cells will migrate then proliferate and form a lumen tube, after that, other vascular cells that are adjacent will be connected in the wound area [38]. By observing the formation of new blood vessels between the healthy group and the wounded group, there was an significant difference (α = 0.00) in the red laser treatment group compared to other treatment groups, including the healthy and wounded groups. The increase of blood vessels is related to new blood formation which increases with the exposure of the 649 nm laser light and will continue to increase when the laser used has a higher wavelength [40]. Other studies showed that the use of a 660 nm 40 mW laser with 4 J/cm^2^ energy density improved wound healing by increasing neocola genesis, increasing the number of new vessels formed in tissue (neoangiogenesis), and modulating MMP-2 expression. Epidermal MMP-2 overexpression correlates with angiogenic processes [41].

During inflammation, an increase in IL-1β level observed in the wounded group. Furthermore, treatment result significant reduction in IL-1β levels in wounded tissue compared to the positive control group (*p* = 0.00). Another study also demonstrated a significant increase in corticosterone and a significant decrease in IL-1β and IL-6 levels in wounded tissue irradiated with a low energy red laser [37, 42].

In addition, the results indicated an increase in the expression of collagen-1α in each treatment group compared to the wounded Wistar rats control group. The collagen-1α expression on the red laser treatment group was the highest compared to the wounded group and other treatments. These results are similar to other studies that showing an increase in type 1 collagen occurred from ligament injury up to the fourth week until the amount of collagen was normal [43].

**Fig. 6:**
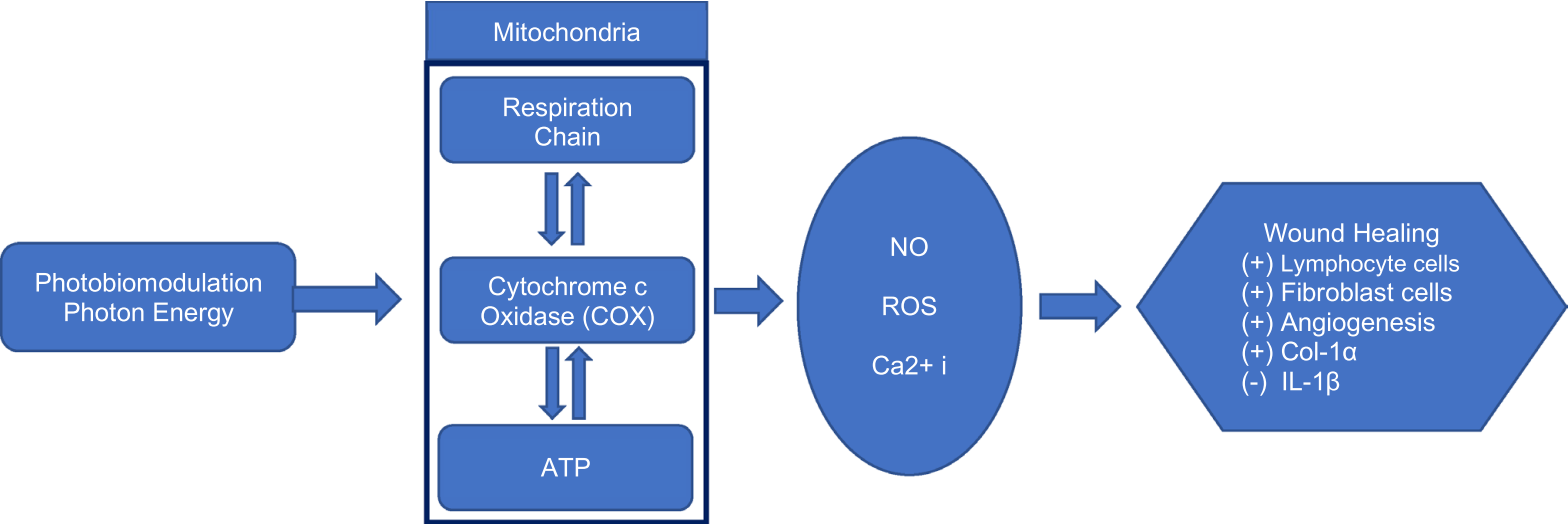
The outline of the PBM process for wound healing

The PBM process affecting wound healing is illustrated in Fig. 6. Based on the results of the study, the red laser PBM treatment showed better results compared to the other therapy groups. The results are confirmed with the study by Dancakova (2014) which concluded that PBM can increase the level of ATP in superficial tissue and the brain, then releases Nitric Oxide (NO) and Reactive ROS and intracellular calcium, which are the initial stages of the wound healing process [44, 45]. PBM therapy increases the activity of Cytochrome C Oxidase (COX) in mitochondria which plays a role in photon energy absorption and ATP synthesis [44]. COX is the basis of the mitochondrial respiratory chain which has an important role in producing energy. Increased activity of the mitochondrial respiration chain will increase ATP synthesis in brain tissue, releasing NO, ROS, and intracellular calcium resulting in wound healing and preventing dead tissue by activating transcription factors, antiapoptotic, antioxidants, and proliferation gene products [26, 46].

The results of this study revealed that red laser treatment causes an increase of fibroblast cells and expression level of Col-1α which then also increase the tensile strength of the tissue [17, 43]. Another study showed an improved wound healing by laser PBM therapy at 532–1064 nm with 0.09 to 90 J/cm^2^ energy density, also resulting significant wound improvement with 632.8–830 nm wavelength, and 1 to 5 J/cm^2^ energy density [46].

In a similar study, Ribeiro et al. (2009) reported that the group of rats given 660 nm red laser PBM therapy showed higher number of myofibroblasts cell than the group without exposure [36]. Fibroblasts are the main cells in the proliferation phase that provides the extracellular matrix as a framework for keratinocyte migration [38]. Denser fibroblasts help the formation of a more compact and denser extracellular matrix, hence initiating the epithelialization process by keratinocytes [16].

During the inflammatory phase, the administration of red laser treatment group is found to increase the number of lymphocyte cells, but no significant difference in IL-1β expression compared to the control group (*p* = 0.99) and the doxycycline group (*p* = 0.05). The proliferation phase is indicated by increase in fibroblast cells, the formation of new blood vessels, and an increase in the expression of collagen protein. Thus, the red laser PBM can consistently assist the healing process based on the results of histopathological and immunohistochemical tests. The proliferation process of wound healing can occur earlier if red laser PBM therapy is applied [45, 46].

The red laser has deeper penetration into the tissue so that it has a significant effect on wound healing. Blue laser usually used for antimicrobial therapy [13, 14], but it is also able to show a positive effect on wound healing even though has lower penetration into the tissue [47]. The other studies indicated the effect of blue light on NO metabolism, an important mediator in wound healing [35]. The optical window for the PBM effect is in the wavelength range of 600–1300 [45]. While the endogenous porphyrin absorption area which provides antimicrobial effect is in the range 400—700 nm with a soret band peak at 402 nm [48]. Blue laser irradiation is capable of releasing NO from NO–Hb complexes, increasing local tissue perfusion and releasing NO from mitochondrial complexes, activating growth factors, and inducing endothelial cell migration, adhesion and proliferation [49].

## Conclusion

Based on the histopathological test results, red laser diode beam exposure with 649 nm wavelengths and 4.0 J/cm^2^ energy density, 42 s exposure time in post-tooth extraction wounds significantly increased lymphocyte cells, fibroblast cells and the formation of new blood vessels. Meanwhile, immunohistochemical tests results showed an increase in the expression of the Col-1α protein which plays a role in the formation of collagen as new tissue formation after damage occurred, as well as a decrease in the level of IL-1β expression. Blue laser 403 nm with energy density 8.0 J/cm^2^, 44 s exposure time, is able to show a positive effect on wound healing even though has lower penetration into the tissue. Therefore, we can conclude the red diode laser 649 nm has been shown to accelerate the process of proliferation in wound healing post molar extraction based on histopathological and immunohistochemical test results.

## Data Availability

The datasets analyzed during the current study are available from the corresponding author on reasonable request.
